# Metabolism meets immunity: TCA-cycle activation protects the vulnerable newborn

**DOI:** 10.1038/s44321-026-00462-0

**Published:** 2026-06-08

**Authors:** Tineke Vanderhaeghen, Claude Libert, Jolien Vandewalle

**Affiliations:** 1 VIB Center for Inflammation Research, VIB, Ghent, Belgium; 2 Department of Biomedical Molecular Biology, Ghent University, Ghent, Belgium

**Keywords:** Metabolism, Microbiology, Virology & Host Pathogen Interaction

## Abstract

T. Vanderhaeghen, C. Libert and J. Vandewalle discuss the study by Wu et al, in this issue of *EMBO Mol Med*, that identifies tricarboxylic acid (TCA) cycle metabolites as key modulators of early life infection outcomes.

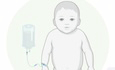

## Teaser

Preterm neonates are highly susceptible to infections which can easily develop into sepsis. Neonatal sepsis remains a major global burden, as no clear pathophysiological characteristics and biomarkers are defined, diagnosis remains challenging, and therapy is limited to antibiotics and general supportive care. Furthermore, how the immune system interacts with the energy metabolism of preterm infants upon infection is poorly understood. In this issue, Wu et al (2026) demonstrate a clear link between systemic energy metabolism and neonatal defense mechanisms during infection. The authors identified tricarboxylic acid (TCA) cycle metabolites as key regulators of immune function associated with reduced infection burdens in early childhood. Boosting TCA cycle activity by providing parenteral nutrition (PN) with galactose or glucogenic amino acids improved glucose homeostasis, enhanced pathogen clearance, and dampened excessive inflammation, ultimately reducing lethality in a piglet neonatal sepsis model. Together, these findings propose therapeutic strategies to improve the outcome of preterm newborns.

Preterm newborns are extremely vulnerable to infections, which can rapidly progress into neonatal sepsis. Since the neonatal period represents the highest lifetime risk period for sepsis, it carries a huge medical, economic, and social burden. It remains a major global challenge, with an estimation of up to 5 million cases and 800,000 deaths yearly (Strunk et al, 2024). In contrast to sepsis in older children and adults (Singer et al, 2016), no universally accepted definition for neonatal sepsis exists. Diagnosis remains challenging because neonatal sepsis lacks well-defined pathophysiological hallmarks and reliable biomarkers. Furthermore, the limited blood volumes obtainable from symptomatic newborns reduce the sensitivity of cultures from normally sterile body fluids (Singer et al, 2026). Besides difficult diagnosis, neonatal sepsis therapy is limited to timely antibiotic treatment and general supportive care, e.g., adequate oxygenation and perfusion, thermoregulation, fluid homeostasis, balanced acid and base status, and striving towards normoglycemia.

Although the high infection rate is generally attributed to their immature immune system, clinical trials focusing on immunomodulation have not resulted in improved outcomes, underscoring the need for new innovative therapeutic strategies (Schüller et al, 2018; Strunk et al, 2024). One of the potential reasons for these failures is the traditional translational pipeline typically used in sepsis, from in vitro and in vivo—mainly murine— studies to clinical validation. Although animal models have substantially advanced our understanding of sepsis pathophysiology, many promising preclinical findings have failed to be confirmed in a clinical setting. While animal models remain indispensable, there is a need for stronger emphasis on human-based studies and clinical investigations whenever ethically and practically feasible.

The study by Wu et al published in *EMBO Mol Med* provides a striking example of a successful bedside-to-bench approach. In a cohort of 700 newborn infants (Bisgaard et al, 2013), the authors identified that high levels of TCA cycle metabolites in the plasma are linked with changes in immune cell subsets and reduced infection risks during early childhood (infection burden from birth to 36 months). However, these observational findings could not establish causality or identify the tissues and metabolic pathways underlying these associations. Importantly, the liver is a central organ in neonatal host defence during infection, integrating systemic metabolic, immunological, hormonal, and nutritional signals. Because studying the hepatic transcriptome and metabolome in newborn infants is ethically and practically challenging, the authors turned to a translational preterm piglet model in which newborn piglets were infused with either *Staphylococcus epidermidis* (*S. epidermidis*) or vehicle control. The associations observed in the human infant cohort were recapitulated in the piglet model: piglets that survived infection displayed higher hepatic TCA cycle metabolite levels than non-survivors, suggesting that preserved hepatic TCA cycle activity protects against infection-induced lethality (Wu et al, 2026).

**Figure 1:**
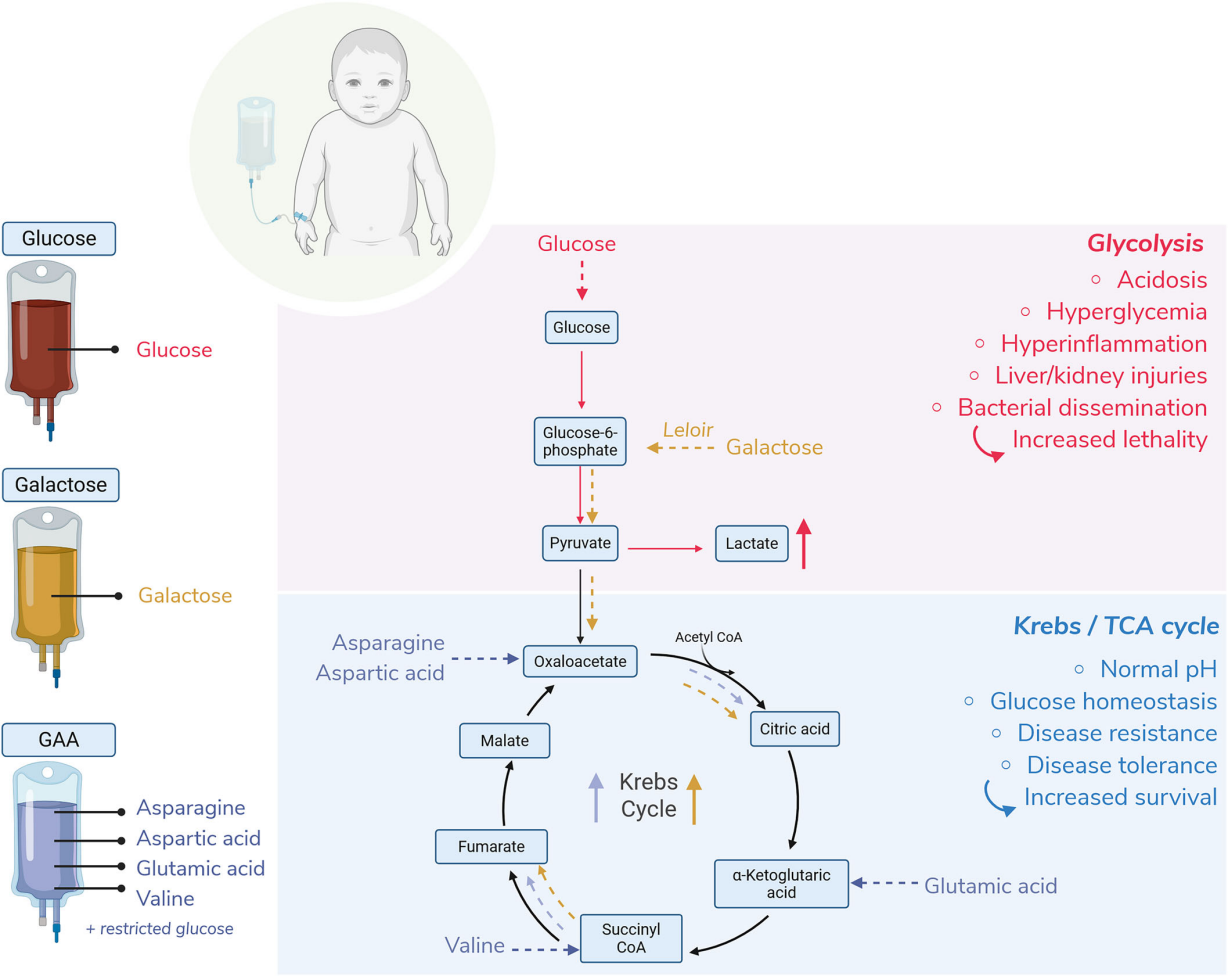
Redirecting energy metabolism from glycolysis to the TCA cycle via parenteral nutrition improves neonatal sepsis outcome. In preterm infants, glucose-rich parenteral nutrition is commonly administered during the first weeks of life. However, this practice skews metabolism to glycolysis instead of oxidative phosphorylation (OxPhos). Increased reliance on glycolysis is associated with acidosis, hyperglycaemia, excessive inflammation, organ injury, and increased susceptibility to infection, thereby aggravating sepsis severity. Replacing glucose with an isocaloric amount of galactose in a preterm pig neonatal sepsis model induces a slower conversion of galactose into glucose-6-phosphate, thereby reducing systemic glycolytic flux while increasing dependence on TCA cycle activity and mitochondrial OxPhos. This metabolic rewiring attenuated the pro-inflammatory activity of immune cells without compromising energy homeostasis or bacterial clearance. Moreover, blood glucose and pH levels remained stable, contributing to reduced lethality. Similarly, supplementation of glucogenic amino acids (GAAs; glutamate, valine, aspartate, and asparagine) together with restricted glucose infusion enhanced TCA cycle activity, as these amino acids enter the cycle either directly or through intermediary metabolites. This intervention promoted normoglycemia, reduced bacterial burden (disease resistance), and preserved blood pH despite comparable bacterial loads (disease tolerance), ultimately providing complete protection against infection-induced mortality in piglets. Created in BioRender. https://BioRender.com/7g8l5pw.

To causally demonstrate the protective effects of TCA metabolites, the authors enhanced TCA cycle activity by replacing glucose in PN with galactose or glucogenic amino acids (GAAs, containing glutamate, valine, aspartate, and asparagine) (Fig. 1). Preterm neonates are typically provided with glucose-rich PN during the first weeks of life to prevent hypoglycaemia. However, this nutritional strategy increases the risk of hyperglycaemia, which is linked to a higher infection rate and sepsis severity (Moltu et al, 2021). Substituting glucose with galactose or GAA-based nutrition improved disease resistance and/or tolerance by respectively enhancing pathogen clearance or maintaining host health despite comparable bacterial loads, resulting in improved survival (Wu et al, 2026). Numerous sepsis animal models have been developed, e.g., caecal ligation and puncture, systemic bacterial challenge in rodents; unfortunately, studying the effects of PN in newborn rodents remains challenging. Therefore, preterm pigs form a unique, premature animal model allowing PN via an umbilical catheter and mimicking cellular and clinical responses to an infection, as found in preterm infants (Muk et al, 2022).

Since Wu et al have indicated a clear association between TCA cycle metabolite levels in the plasma of pregnant women and the infection rate in preterm newborns, it might be of great interest to measure these metabolites during pregnancy and develop new supplementation strategies in order to maintain sufficient levels of these TCA cycle metabolites at birth and reduce neonatal infection rates. Micronutrient supplementation has already been shown to reduce low birthweight births and neonatal mortality, but more interventions are necessary to achieve global targets (Hofmeyr et al, 2023). Furthermore, since biomarkers for neonatal sepsis are lacking (Strunk et al, 2024), TCA cycle metabolites might serve as potential biomarkers for neonatal sepsis, as good TCA cycle activity is required for the rapid growth and development of preterm infants, and high levels of TCA cycle metabolites are linked with a reduced infection risk. Here, Wu et al have clearly shown the therapeutic potential of PN supplementation with galactose or GAAs in preterm piglets upon *S. epidermidis* infection (Wu et al, 2026). It might be an added value to study the effects of other neonatal pathogens in these preterm piglets, to verify the applicability of their findings in a broader context. If promising, these results would encourage to test these interventions in a clinical setting. Standard neonatal PN already contains GAAs as part of balanced amino acid formulations, but not at ratios specifically designed to stimulate the hepatic TCA cycle activity and prevent glycolysis, highlighting the need for further investigations. In contrast, supplementation of PN with galactose, thereby more closely mimicking the carbohydrate composition of maternal milk, is not standard clinical practice, although it has recently been proposed as a strategy to improve neurodevelopmental outcomes in preterm neonates (Panfoli, 2025). Taken together, this study of Wu et al provides clinically relevant strategies aimed to protect preterm infants from infections at the level of energy metabolism and infection defence strategies.
